# Supplementary material for: A Meta-Analysis on the Association between Peptic Ulcer Disease and COVID-19 Severity
Source: Vaccines (Basel). 2023 Jun 11;11(6):1087. doi: 10.3390/vaccines11061087 (PMC10304706; doi:10.3390/vaccines11061087)
Supplement: Supplementary file 1 [file vaccines-11-01087-s001.zip › vaccines-2318989-supplementary.pdf]

Table S1. Quality assessment of eligible studies.

Study	Selection				Comparability	Outcome			Score
	Representativeness of the exposed cohort	Selection of the non-exposed cohort	Ascertainment of exposure	Demonstration that outcome of interest was not present at start of study	Comparability of cohorts on the basis of the design or analysis	Assessment of outcome	Follow-up long enough for outcomes to occur	Adequacy of follow-up of cohorts	
Kabarriti R	★	NA	★	NA	NA	★	★	★	5
Oh TK	★	★	★	★	★	★	★	★	8
Navaratnam AV	★	NA	★	NA	NA	★	★	★	5
Cho SI	★	NA	★	★	NA	★	★	★	6
Oh TK	★	★	★	★	★	★	★	★	8
Gray WK *	★	NA	★	NA	NA	★	★	★	5
Choi YJ	NA	NA	★	NA	NA	★	★	★	4
Meis-Pinheiro U	NA	NA	★	NA	NA	★	★	★	4
St Sauver JL	NA	NA	★	NA	NA	★	★	★	4
Yang X	★	★	★	★	★	★	★	★	8
Aziz F	★	NA	★	NA	NA	★	★	★	5
Meister T	★	NA	★	NA	★	★	★	★	6
Rainer L	★	★	★	NA	★	★	★	★	7
Piskac Zivkovic N	NA	NA	★	NA	NA	★	★	★	4
Yoshida Y	★	NA	★	NA	NA	★	★	★	5

* indicates that the combined value was calculated based on data from subgroups.
